# Analysis of clinical features and inflammatory-related molecules with the disease in acute infectious urticaria

**DOI:** 10.1007/s00403-023-02564-y

**Published:** 2023-02-28

**Authors:** Zhezhang Liu, Lina Al-Quran, Jianbo Tong, Xianwei Cao

**Affiliations:** 1 Department of Dermatology, The First Affiliated Hospital of Nanchang University, Nanchang, Jiangxi China; 2 Institute of Dermatology, Jiangxi Academy of Clinical Medical Sciences, No. 17 Yongwaizheng Street, Nanchang City, Jiangxi Province China

**Keywords:** Acute infectious urticaria, CCL8, Neutrophil, MPO

## Abstract

Acute infectious urticaria, a subset of acute urticaria, with severe persistence wheals and systemic symptoms, response well to corticosteroids treatment in combination with antibiotics. The exact pathogenic mechanisms are not fully understood. In this study, we aim to analyze the different clinical features, compare the level of neutrophil activation, and investigate the expression of inflammatory related cytokine in patients with acute urticaria and acute infectious urticaria. Eighteen patients with acute infectious urticaria and eighteen patients with acute urticaria were included in this study. We analyzed the difference between the clinical features and the serum expressions of pro-inflammatory factors in the two groups, then examined the levels of inflammation-associated cytokines before and after treatment of acute infectious urticaria. Hematoxylin & eosin (HE) staining and immunohistochemistry (IHC) were used to further study the relationship between neutrophil and neutrophil-derived Myeloperoxidase (MPO) of lesions in the two groups. The expression levels of C-reactive protein (CRP), D-dimer, interleukin 6 (IL-6), IL-8 and chemokine ligand 8 (CCL8) in serum were significantly higher in acute infectious urticaria than acute urticaria. In acute infectious urticaria, the serum expression levels of CCL8 were significantly decreased after the treatment, a significant correlation observed between CRP levels and IL-6, both CCL8 and CRP were positively correlated with neutrophil granulocytes. Neutrophils infiltration were not observed by HE stains in two groups, but in IHC stains we found a positive expression of MPO in acute infectious urticaria lesions. Elevated neutrophil in the serum, which is associated with the levels of IL-8 & CCL8, and positively expressed MPO in lesions, may be involved in the pathogenic mechanism of acute infectious urticaria.

## Introduction

Acute urticaria is a common condition with itching, wheals, or angioedema caused by various etiologic factors [1]. A group of severe acute urticaria, appears as multiple and sustained wheals partially ring-shaped, which are resistant to conventional treatment with antihistamines and present with symptoms such as fever, leukocytosis, high levels of CRP, and good therapeutic response to antibiotics, which may be caused by bacterial infection [2]. In Japan, the concept of acute urticaria caused by a bacterial infection without determined infectious foci is considered to some extent to be acute infectious urticaria. Previous studies suggest a diagnostic criterion for acute infectious urticaria that included poor response to antihistamines [3, 4], fever higher than 37 °C with severe diffuse wheals, and more than two of the following three laboratory abnormalities (including leukocytosis greater than 10,000/ mm^3^, neutrophilia greater than 70% of total leukocyte count, elevated CRP greater than 0.5 mg/dL).

Acute-phase response (APR), following infections, tissue injury and inflammatory processes, are a local and systemic coordinated reaction [5]. In addition, there is a relation between APR and activation of coagulation and fibrinolysis in urticarial [6, 7]. Chemokines are chemotactic cytokines that induce directional movement and activation of leukocyte subsets [8]. Chemokines expressed in the skin contribute to the development and maintenance of allergic processes through the coordinated recruitment and activation of leukocytes and resident cells at the site of allergic inflammation [9, 10]. Triggering of acute infectious urticaria by various infections has been thought for many years.

Studies have shown that neutrophils may migrate to the infection site due to the influence of chemokines [11], however, it is unclear whether neutrophils are involved in lesions of acute infectious urticaria. MPO is a marker of neutrophil activation in serum [12, 13], an increase of the level of MPO triggers a damage of the vascular endothelial, which result in an edema of the tissues [14–18].

Acute infectious urticaria is a specific form of acute urticaria whose pathogenesis is not yet clear. The aim of this study was to analyze and compare the different characteristics of acute infectious urticaria and acute urticaria, to investigate the expression of cytokines and chemokines that may be involved, and to work out the interaction between them in acute infectious urticaria.

## Methods

### Subjects

This study was approved by the ethics committee of the First Affiliated Hospital of Nanchang University. Patients were fully informed about the study and asked for their consent before participating. Thirty-six patients (Acute infectious urticaria *n* = 18, male/female 6/12, mean age 30.50 (21.25, 42.75) years. Acute urticaria *n* = 18, male/female 8/10, mean age 22.50 (17.75, 42.50) years). Age- and sex-matched patients were recruited for the acute urticaria group. The diagnosis of acute urticaria was based on the new EAACI/GA2LEN/WAO/EDF guidelines for patients with an infection sign [1]. The inclusion criteria of acute infectious urticaria were based on these literatures [2–9]. We collected clinical information and serum samples from the patients, as well as skin lesion samples from four of them.

At the beginning of the study, subjects detailed medical history was obtained by general examination to exclude all underlying diseases. Patients with a total history of more than 6 weeks, urticarial vasculitis, previous or current autoimmune or inflammatory disease, and use of corticosteroids or immunosuppressants in the 7 days before enrollment in the study were excluded. The following laboratory tests were examined: complete blood count, urinalysis, stool analysis, erythrocyte sedimentation rate (ESR), CRP, liver functions, serum creatinine and coagulation analysis, rheumatoid factor, antistreptolysin O (ASO), complement 3 (C3), complement 4 (C4), antinuclear antibodies, and antithyroid microsomal antibodies were performed in all patients.

### Blood collection

Venous blood samples (5–10 mL) were collected in the morning in pyrogen-free Vacutainer tubes under sterile conditions. Serum was obtained from freshly collected, rapidly centrifuged samples. Patient serum samples were rapidly frozen at − 80 °C and stored until processing.

### Serum cytokine analysis in Cytometric Bead Array

Inflammatory-related cytokines were quantified in stored serum using a multiplex assay according to the manufacturer's instructions (CEGER Cytometric Bead Array (CBA) Human Cytokine kit; CEGER Biosciences, Jiangxi, CHINA). Concentrations were calculated using various cytokine patterns at standard concentrations. All samples were analyzed using BECKMAN COUNLTER flow cytometer (DXFLEX), and results were expressed in pg/mL using Kaluza Analysis Software (BECKMAN Biosciences).

### Serum cytokine analysis in Elisa

Cytokines and chemokines were measured in stored serum using a commercially available solid-phase enzyme-linked immunosorbent assay (ELISA) kit (4A Biotech, Beijing, CHINA) according to the manufacturer's instructions. The fully automated microplate analyzer (Thermo Scientific, VARIOSKAN LUX, USA) was used for the assays. Skanlt RE software is used for data processing and standard curve generation, and then automatically read the concentration of the assays in the unknown samples and control.

### Hematoxylin and eosin staining

To confirm neutrophilic infiltration into the lesions of the patients with urticaria, skin samples were collected and fixed in 10% formalin, tissue samples from two patients with acute urticaria and two patients with acute infectious urticaria. Paraffin embedded sections (4 μm) were stained with hematoxylin and eosin. The stained slides were subsequently evaluated under an inverted microscope (ZEISS, Axio Lab.A1 + ERC5S, GER).

### Immunohistochemical staining

Tissue samples from two patients with acute urticaria and two patients with acute infectious urticaria. Paraffin embedded sections (4 μm) were stained with primary antibodies: Anti-Myeloperoxidase (1:1000 dilution, Abcam), the sections were treated with protein blocking solution and primary antibodies for 2 h at room temperature. The sections were then incubated with biotinylated secondary antibodies after several rinses in phosphate-buffered saline (PBS). Secondary antibody is Goat Anti-Rabbit IgG H&L (HRP) (1:500 dilution, Abcam). All sections were evaluated by conventional light microscopy (ZEISS, Axio Lab.A1 + ERC5S, GER).

### Statistics

Anderson–Darling test, D'Agostino & Pearson test, Kolmogorov–Smirnov, and Shapiro–Wilk test were used to assess the normal distribution of all data. Mean ± standard deviation was used to express continuous variables with normal distributions. Medians and interquartile ranges were used to describe those with non-normal distributions. Student's *t* test or Mann–Whitney *U* test used to analyze the differences between two groups. One-way analysis of variance was used to compare the differences between more than two groups. Spearman correlation coefficient is used to test the correlation of continuous variables. Statistical significance was determined by two-sided P values of less than 0.05 for all tests. Data were analyzed using SPSS version 22.0 (SPSS Inc, Chicago, IL, USA) and GraphPad Prism version 9 (GraphPad Software, San Diego, CA, USA).

## Results

**Fig. 1:**
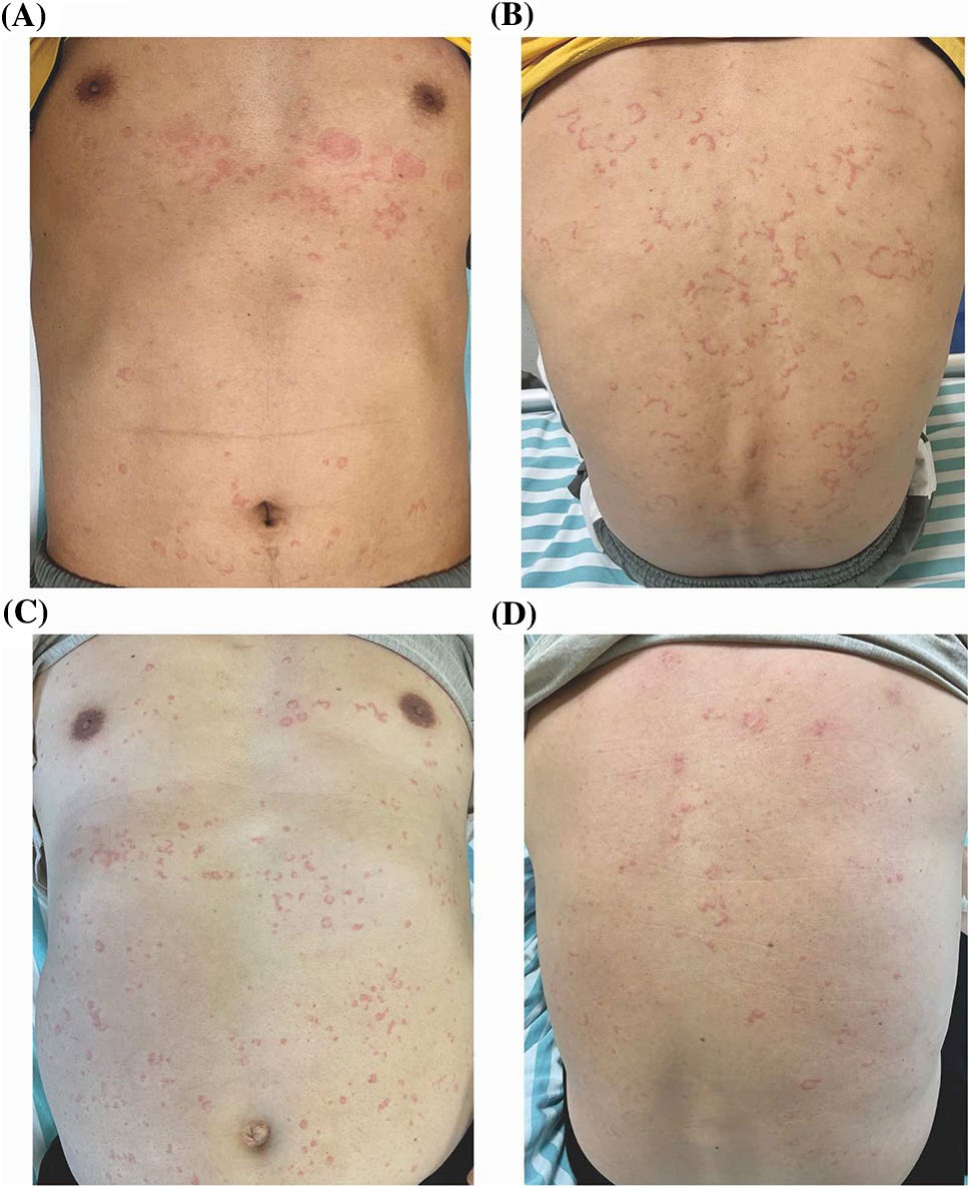
Cutaneous manifestation of the representative case. **A**–**D** Multiple wheal which last longer than common acute urticaria were seen on the whole body, commonly ring-shaped, with the skin color tinged with dark red

This study included 36 patients with acute infectious urticaria and acute urticaria, all matched for age and sex (Table1). There are Cutaneous manifestation in two representative cases (Fig. 1).

**Table 1:** Demographic and clinical characteristics of the subjects

Case	Sex	Age (year)	Leukocyte (10^9^/L)	Neutrophil (10^9^/L)	Neutrophil (%)	CRP (mg/L)	D-dimer (mg/L)	Temperature (℃)	Therapy
1	F	19	19.62	17.78	90.6	58.26	1.21	39.0	D + C $\to$ D + MO
2	F	26	22.54	20.88	92.7	107.73	1.93	38.10	D + A $\to$ D + A + M
3	F	15	31.29	29.72	95.0	93.56	0.87	37.7	M + C
4	F	45	25.81	23.62	91.5	179.96	9.76	37.6	M + L $\to$ M + A
5	F	25	13.73	12.5	91.1	35.58	1.78	37.5	MP $\to$ M + L
6	F	18	13.3	10.98	82.6	27.67	0.36	38	M + L
7	F	18	15.86	13.89	87.7	10.02	3.29	37.5	M $\to$ M + L
8	M	34	14.46	13.58	94.0	92.43	0.81	38	M + L $\to$ M + C
9	M	47	10.96	9.5	86.6	1.65	0.26	37.5	M + L $\to$ M + C
10	F	22	28.83	27.19	94.3	155.55	1.86	38	D + L $\to$ D + O
11	M	53	11.08	9.89	89.3	14.48	13.4	37	M + L
12	M	42	11.29	10.27	91.0	139	1.54	39	M + L
13	M	30	13.51	11.4	84.3	103.38	9.21	38.3	M + L
14	F	31	9.77	8.83	90.3	24.35	0.26	37.5	M + L
15	F	34	21.76	20.8	95.5	57.85	4.58	37.5	M + L $\to$ M + C
16	F	27	15.65	14.77	94.4	17.71	2.34	37.5	M + L
17	M	31	13.55	12.01	88.6	197.75	0.77	38.1	M + L
18	F	56	11.54	9.39	81.4	33.22	0.71	37.5	M + L
19	M	28	3.82	1.79	46.9	0.68	0.16	36.5	H
20	F	19	6.63	5.69	85.8	3.49	3.65	36.7	H
21	M	14	7.39	3.93	53.2	5.86	0.27	36.6	H
22	F	58	6.43	5.49	85.3	8.00	0.38	37.0	H
23	M	34	6.24	4.04	64.7	4.37	0.40	37.0	H
24	M	55	5.94	3.69	62.3	2.37	1.49	36.5	H
25	M	44	9.34	7.40	79.2	0.10	0.20	36.5	H
26	M	12	7.98	6.38	79.9	6.50	2.73	36.4	H
27	F	32	6.43	5.59	87.0	0.89	0.04	37.0	H
28	F	19	11.32	5.12	45.2	0.40	0.05	37.1	H
29	F	48	9.48	6.0	63.3	1.15	0.59	36.6	H
30	M	20	8.29	5.82	70.4	17.31	0.58	36.3	H
31	F	13	8.99	5.19	57.8	2.32	11.70	36.5	H
32	F	21	9.57	7.51	78.5	0.01	0.26	36.7	H
33	M	18	5.04	2.44	48.4	4.23	0.23	36.6	H
34	F	24	11.68	7.02	60.2	0.03	0.13	36.8	H
35	F	42	6.77	4.0	59.1	2.65	1.34	36.6	H
36	F	17	6.55	3.19	48.8	6.30	0.32	37.0	H

*CRP* C-reactive protein, *C* Ceftriaxone, *A* Azithromycin, *D* Dexamethasone, *M* Methyprednisolone, *L* Levofloxacin, *MO* Moxifloxacinmo, *H* Antihistamines

Case (1–18): Acute infectious urticaria patient; Case (19–36): Acute urticaria patients

Normal value: Leukocyte(10^9^/L): 3.5–9.5, Neutrophil(10^9^/L): 1.8–6.3, Neutrophil(%):40–75, CRP (mg/L): 0–8, D-dimer (mg/L): 0–0.243, Temperature (℃): 36–37

### Clinical features in acute infectious urticaria

**Fig. 2:**
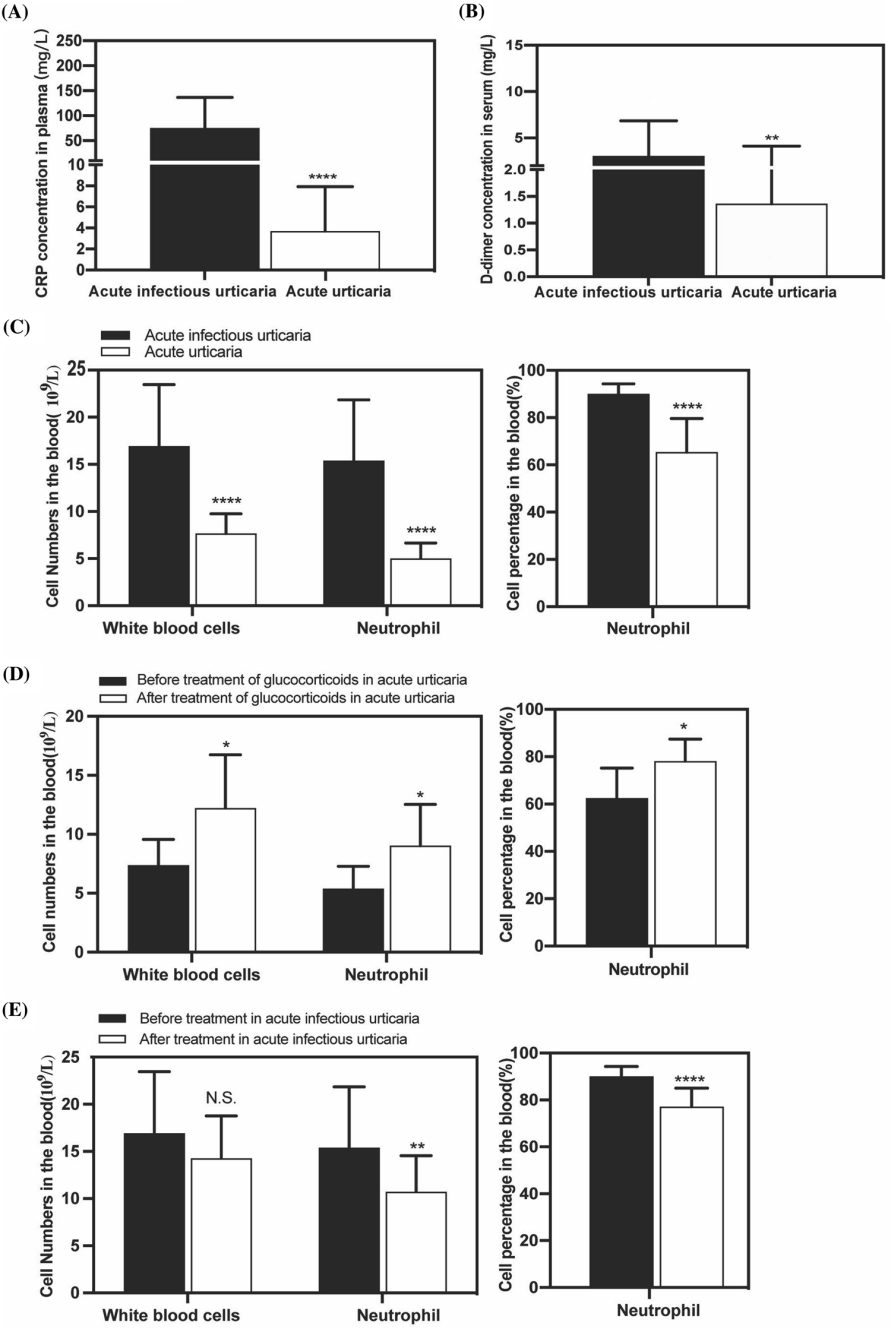
The clinical features in acute infectious urticaria. **A**–**B** The serum concentrations of CRP and D-dimer in acute infectious urticaria and acute urticaria. **C** Comparison of cell population and neutrophil percentage between acute infectious urticaria and acute urticaria. **D** Comparison of cell population and neutrophil percentage before and after treatment of glucocorticoids in acute urticaria. **E** Comparison of cell population and neutrophil percentage before and after treatment in acute infectious urticaria. *P < 0.05; **P < 0.01; ***P < 0.001; ****P < 0.0001

Serum expression levels of CRP and D-dimer were significantly higher in acute infectious urticaria patients as compared with the acute urticarias patients (median (mg/l): 58.06 (22.69, 115.5) vs 2.51 (0.61, 5.97), *P* < 0.0001; median (mg/l): 1.66 (0.755, 3.613) vs 0.35 (0.19, 1.378), *P* < 0.01, respectively) (Fig. 2A, B). White blood cell count, neutrophil count, and the percentage of neutrophils were significantly higher in acute infectious urticaria as compared with acute urticarial (*P* < 0.0001, *P* < 0.0001, *P* < 0.0001, respectively) (Fig. 2C). White blood cell count, neutrophil count and the percentage of neutrophil were significantly higher after glucocorticoid’s treatment than before the treatment in acute urticarial (*P* < 0.05, *P* < 0.05, *P* < 0.05, respectively) (Fig. 2D). Neutrophil count and the percentage of neutrophil were significantly higher before treatment as compared after the treatment in acute infectious urticarial (*P* < 0.01, *P* < 0.0001, respectively) (Fig. 2E).

### Serum levels of cytokine by CBA in acute infectious urticaria and acute urticaria

**Fig. 3:**
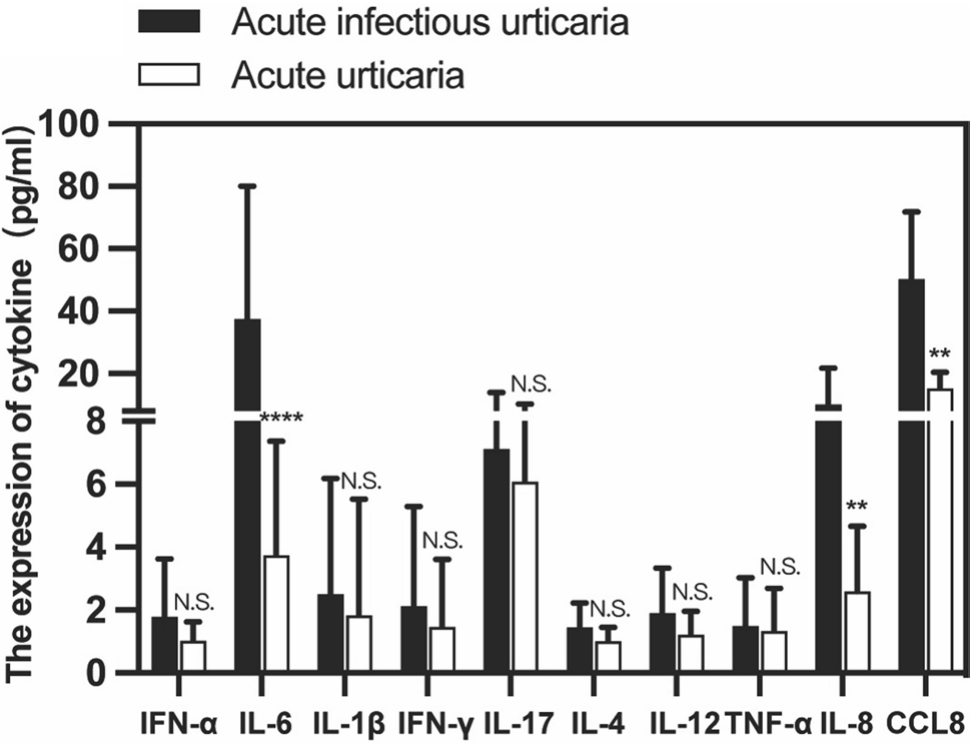
Serum levels of different cytokines between acute infectious urticaria and acute urticaria. The cut-off value of cytokines (pg/mL): IFN-α: (0–8.5), IL6: (0–5.3), IL 1Beta: (0–12.4), IFN-γ: (0–7.42), IL17:(0–20.6), IL12:(0–3.4), TNF-α: (0–4.6), IL8:(0–53.09), CCL8:(0–32.6) *P < 0.05; **P < 0.01; ***P < 0.001; ****P < 0.0001

We selected (IFN-α, IL-6, IL-1β, IFN-γ, IL-17, IL-4, IL-12, TNF-α, IL-8 and CCL8) cytokines and measured in serum of acute infectious urticaria and acute urticaria by CBA. The serum level of IL-6 level was significantly higher in acute infectious urticaria than in acute urticaria (*P* < 0.0001). IL-8 levels in acute infectious urticaria were higher than acute urticaria (*P* < 0.01), but the concentration of IL-8 was within the normal limits. The serum level of CCL8 level was significantly higher in acute infectious urticaria than in acute urticaria (*P* < 0.0001).The serum level of TNF-α, IL-1β, IL-12, IFN-γ and IFN-α did not differ significantly between the acute infectious urticaria and acute urticarial (Fig. 3).

### Serum levels of cytokines by Elisa in acute infectious urticaria before and after treatment

**Fig. 4:**
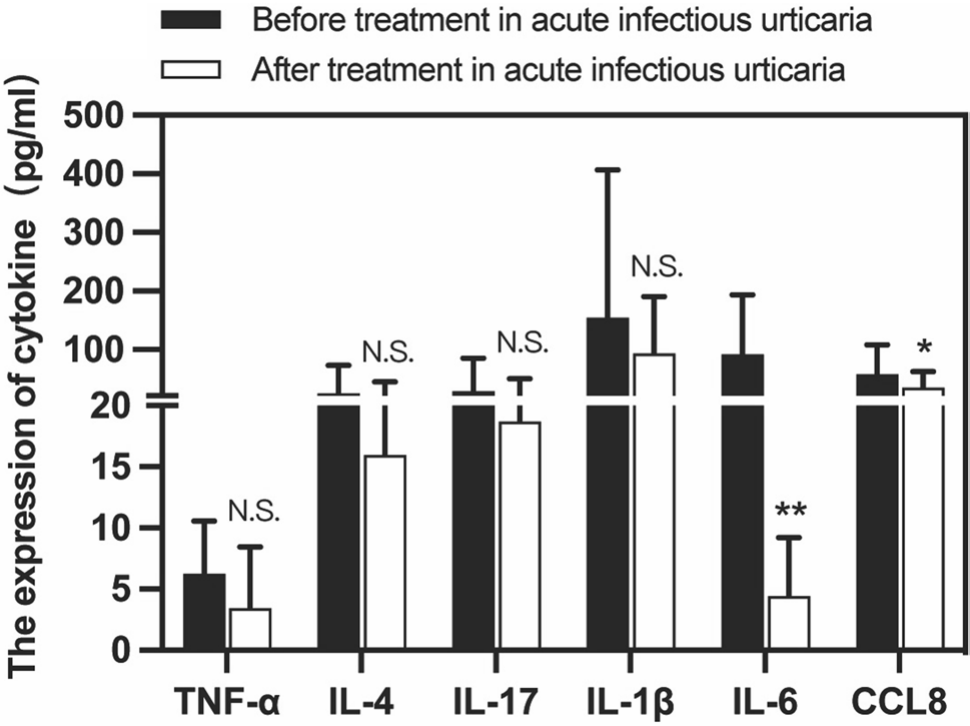
Serum levels of different cytokine in acute infectious urticaria before and after treatment. *P < 0.05; **P < 0.01; ***P < 0.001; ****P < 0.0001

We selected cytokine (TNF-α, IL-4, IL-17, IL-1β, IL-6 and CCL8) and measured in serum of acute infectious urticaria patients before and after treatment by Elisa. The results showed that the serum levels of IL-6 and CCL8 pre-treatment were significantly higher than post-treatment. Moreover, serum levels of IL-1β, TNF-α, IL-4, IL-17, had no significant difference (Fig. 4).

### Correlation

**Fig. 5:**
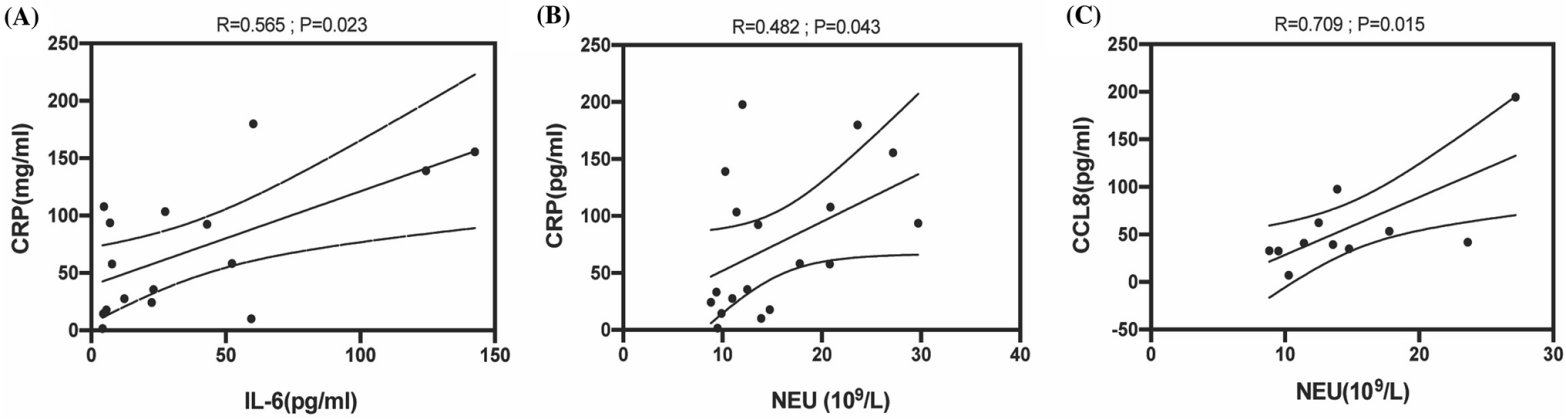
Correlation between the serum levels of CCL8, CRP and Neutrophils, and the serum levels of CRP and IL-6 in acute infectious urticaria. **A** The correlation between IL-6 and CRP concentrations in acute infectious urticaria patients. **B** The correlation between NCU counts and CRP concentrations in acute infectious urticaria patients. **C** The correlation between NCU counts and CCL8 concentrations in acute infectious urticaria patients. *CCL8* chemokine ligand 8, *Neu* neutrophil, *CRP* C-reactive protein, *IL-6* Interleukin-6

The correlations between Neutrophils and both CRP and CCL8 were significant in acute infectious urticaria patients (*R* = 0.482, *p* = 0.043 and *R* = 0.709, *p* = 0.018, respectively). In addition, there were a significant correlation between IL-6 and CRP (*R* = 0.565, *p* = 0.023) (Fig. 5).

### HE staining

**Fig. 6:**
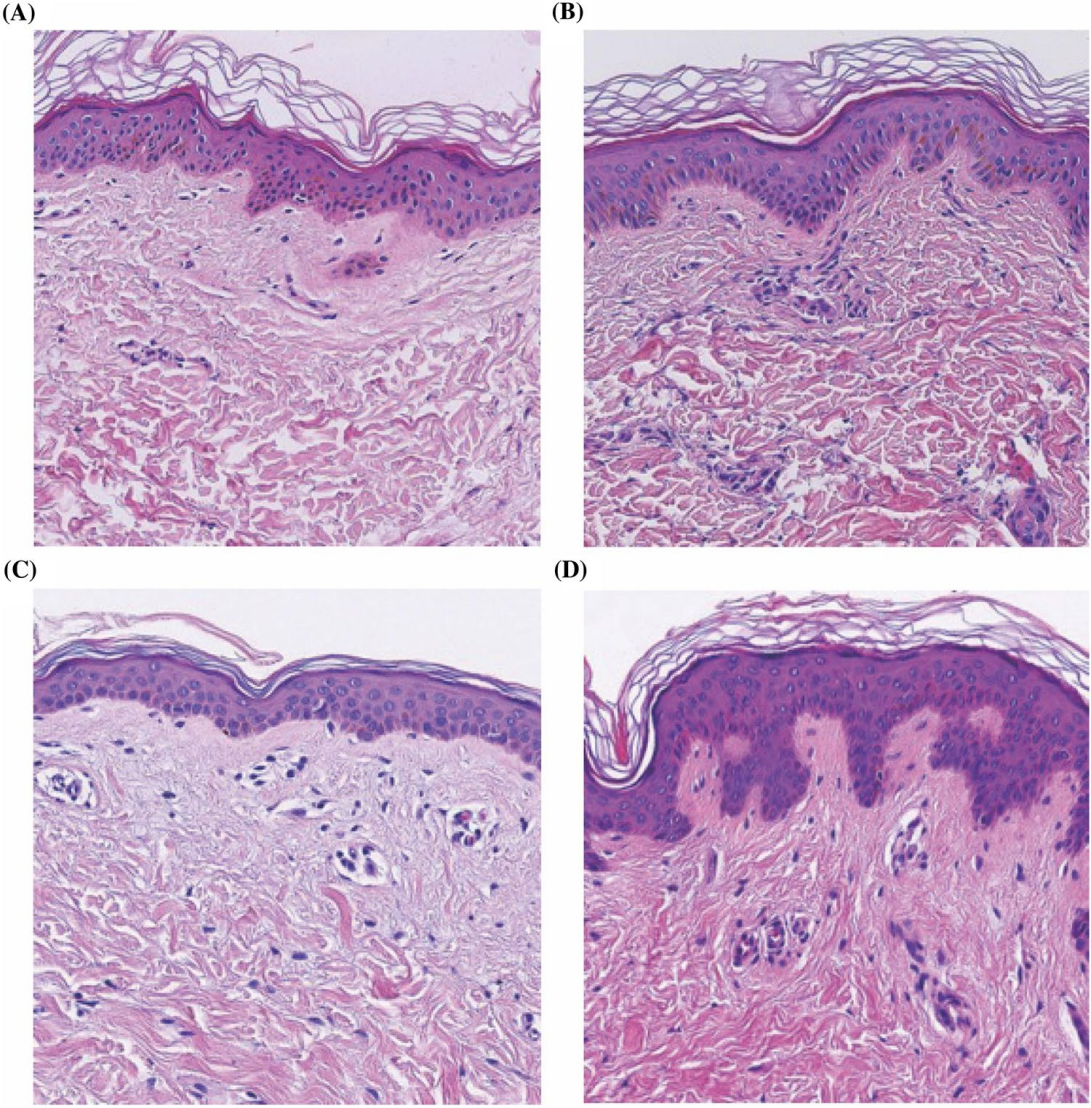
HE staining in acute infectious urticaria (**A**, **B**) and acute urticaria (**C**, **D**). Histological findings of a wheal in two acute infectious urticaria patients and two acute urticaria patients (**A**–**D**). The epidermis showed no significant changes. Dermal edema can be seen in the four cases. Neutrophils infiltrated was not observed (HE, original magnification × 10)

The HE staining showed that the histological characteristics of hives under the microscope were confirmed at the cutaneous lesions in the two groups, primarily showing normal epidermis, sparse infiltration of inflammatory cells around the superficial dermal vessels, lymphocytes, and histiocyte**,** and no obvious neutrophil infiltration. The dermal reticular layer was edematous, and the gap between collagen bundles was enlarged (Fig. 6).

### Immunohistochemistry

**Fig. 7:**
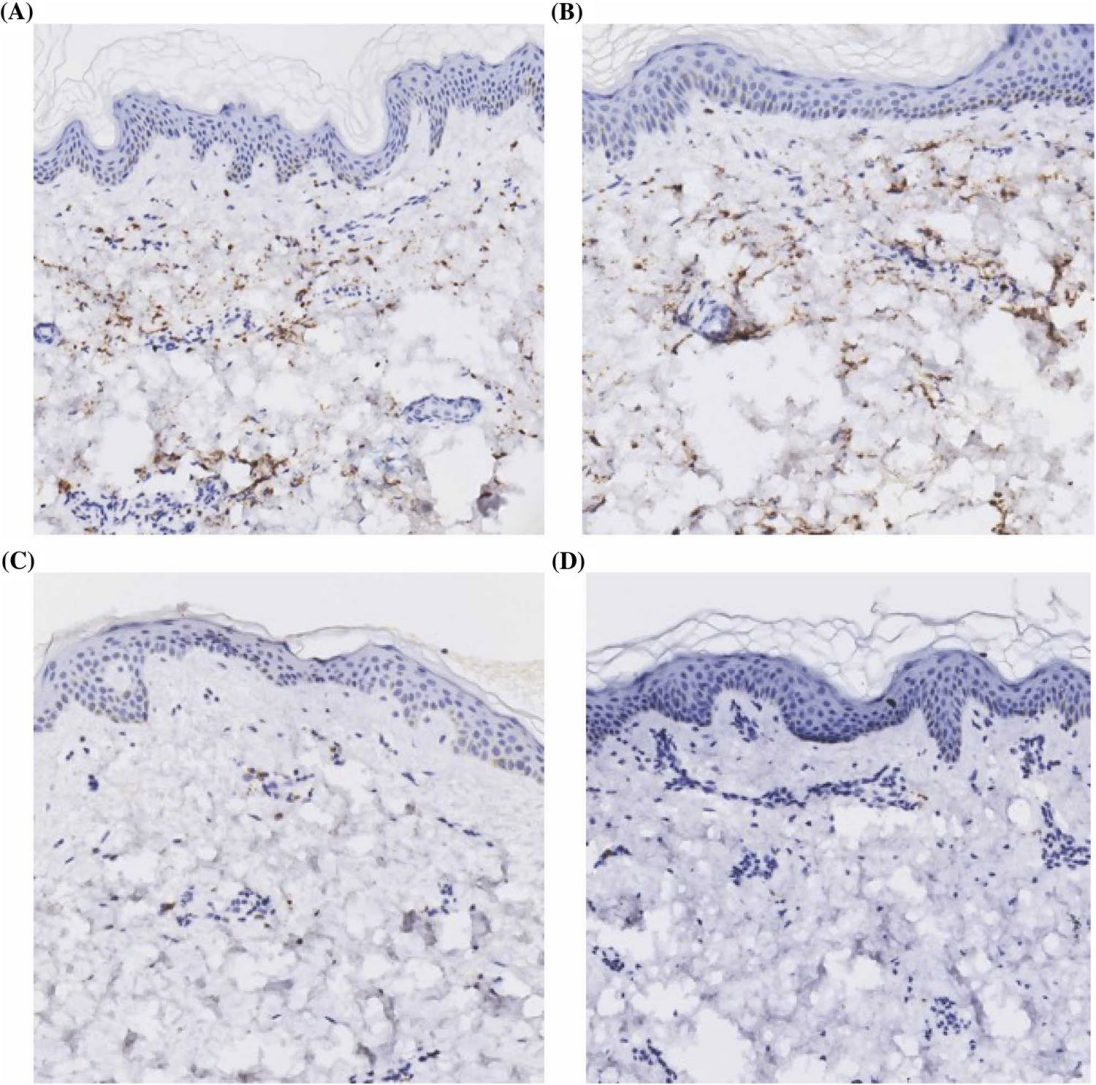
IHC staining in acute infectious urticaria (**A**, **B**) and acute urticaria (**C**, **D**). (IHC, original magnification × 10). **A**, **B** Positive expression and spread throughout the dermis of MPO in two acute infectious patients, primarily in the reticular dermis were showing more visible tissue edema as MPO expression. **C**, **D** The expression of MPO were negative in two acute patients

As a result of immunochemistry, high expression and spread throughout the dermis of MPO, primarily in the reticular dermis were showing more visible tissue edema in acute infectious urticaria patients, as MPO expression were negative in acute urticaria patients (Fig. 7).

## Discussion

We found a part of acute urticaria’s patients caused by infection, especially a bacterial infection, with different clinical management symptoms than those in the common acute urticaria’s patients. Eighteen patients with acute infectious urticaria were based according to the diagnostic criteria which proposed in the previous studies [2–9]. Most of the patients presented with fever, sore throat, and an elevation of white blood cell (WBC), CRP and procalcitonin (PCT) which were supported as a marker of bacterial infection in the previous studies [20]. At the same time, we improved other laboratory tests such as endotoxin, influenza virus throat swabs, bacterial cultures, and fungal cultures, but those tests were limited. All patients were treated with antihistamines and some with dexamethasone, but they did not have any effects. The use of antibiotics had an important role in the treatment. Some of the patients showed a better effect with antibiotics replacement. One patient with acute infectious urticaria presented with fever and chest tightness showed no improvement after histamine and glucocorticoid treatment in the first two days and resulted with a shock on the third day. Addition to the previous therapy we added moxifloxacin, which showed a significant improvement in the patient's condition. In another case, calcitonin gene-positive patient with severe abdominal pain and diarrhea was treated with methylprednisolone and levofloxacin without improvement, however, switching to methylprednisolone and Ceftriaxone immediately alleviated symptoms. According to the clinical features and management therapy in the 18 patients, the diagnosis of acute infectious urticaria was given.

By analyzing the clinical data of our patients, CRP elevation was presented, but D-dimer, and IL-6 which were not included in the previous studies in Tsunoda’s diagnostic urticaria [3], were also elevated in our patients. D-dimer is a marker of fibrinolysis and fibrin turnover [21], which also can be elevated due to a bacterial infection. Asero et al. [22] reported that serum D-dimer levels are higher in patients with severe chronic urticaria than in patients with (moderate/mild) chronic urticaria. An elevation of D-dimer levels is rarely observed in the most acute urticaria, however most of our acute infectious urticaria’s patients were presented with elevated D-dimer levels which were similar to those in Takahashi et al. [19].

Previous studies suggest that IL-6 is important in acute inflammatory responses [23], as during bacterial infection, IL-6 increases rapidly and it can induce APR in the liver, which causes an elevation of CRP levels [24], and this elevation can exacerbate an inflammation and contribute to tissue damage via a complement-dependent mechanism [25]. It has been shown that concentrations of circulating APR biomarkers, IL-6 and CRP, are controlled the extent of the local and systemic inflammatory responses [26, 27], which may directly influence the urticarial response. It is not clear whether the increase of IL-6 and D-dimer is merely an epiphenomenon or may contribute to the pathogenesis of acute infectious urticaria.

We further analyzed the correlated elevation of values, and our results showed a significant positive correlation between CRP and both neutrophils and IL-6, which suggests this correlation regulated by the same mechanisms (APR) in acute infectious urticaria. Grzanka et al. [7] observed that in chronic spontaneous urticaria patients both IL-6 and CRP were positively correlated with D-dimers. Our results showed no correlation between D-dimer and IL-6 or CRP levels in patients with acute infectious urticaria. Therefore, the relation between APR and activation of coagulation/fibrinolysis needs to be investigated.

Studies have confirmed that CCL8 can also be produced by stimuli such as infection and inflammation [28, 29]. At the same time our results showed that the serum levels of CCL8 in acute infectious urticaria were significantly higher than those in acute urticaria. Severa et al. [30] suggested that high expression of CCL8 leads to an increase of neutrophils in peripheral blood. Our results showed that acute infectious urticaria patients were presented with higher CCL8 levels before treatment than after treatment. A significant correlation between CCL8 and neutrophils in acute infectious urticaria were shown. Although the serum level of IL-8 concentrations in both acute urticaria and acute infectious urticaria was in the normal range before the treatment, but the level of IL-8 in acute infectious urticaria were significantly higher than acute urticaria (Fig. 4). Bocheńska-Marciniak et al. [31] demonstrate that IL-8 causes aggregation of neutrophils in vivo and vitro. Alvaro Teijeira et al. [32] suggested that the higher levels of IL-8, the stronger chemotactic effect on neutrophils.

Mast cell degranulation is central to acute urticarial [33], antihistamines are available for the treatment of urticaria, but the majority of patients with acute infectious urticaria were invalid by only antihistamine therapy, which required added systemic steroids and antibiotics to completely suppress wheal formation [4, 19].

Previous study showed a total number of leukocytes and neutrophils significantly increased after glucocorticoids treatment [34, 35], which were similar our results of acute urticaria patients. But in acute infectious urticaria patients they were still receiving glucocorticoids therapy and a significant decrease of neutrophils counts were shown after the treatment, which were different than the results from the previous studies. Therefore, we believe that neutrophils may play a role in acute infectious urticaria.

Whether neutrophils are directly involved in the formation of specific lesions in acute infectious urticaria, two patients with acute infectious urticaria and two patients with acute urticaria were given hematoxylin–eosin staining but neutrophils infiltration were not observed at the lesion site. Surprisingly, we found a positive MPO expression in the acute infectious urticaria skin lesions. MPO is a marker of neutrophil activation in serum [12, 13], according to MPO immunohistochemistry, we recognized a high positive expression of MPO in the dermis, specifically in the reticular dermis which showed an obvious tissue edema in acute infectious urticaria patients, while we didn’t recognize MPO expressions in acute urticaria patients. Studies have shown that MPO can damage endothelial cells and increases vascular permeability which causes an edematous tissue [14, 15]. Therefore, we speculate that MPO may involve in the formation mechanism of the skin lesions in acute infectious urticaria patients.

In acute infectious urticaria, there is no local infiltration of neutrophils in the lesion, but its activation product MPO is highly expressed here, and the specific mechanism still needs to be further explored.

## Data Availability

All data generated or analyzed during this study are included in this article. Further inquiries can be directed to the corresponding author.
